# Carers’ experiences of home enteral feeding: A survey exploring medicines administration challenges and strategies

**DOI:** 10.1111/jcpt.12664

**Published:** 2018-01-19

**Authors:** D. Alsaeed, D. Furniss, A. Blandford, F. Smith, M. Orlu

**Affiliations:** ^1^ School of Pharmacy Department of Pharmacy Practice and Policy University College London London UK; ^2^ UCL Institute of Digital Health University College London London UK; ^3^ UCL Interaction Centre University College London London UK; ^4^ School of Pharmacy Department of Pharmaceutics University College London London UK

**Keywords:** carers, challenges, feeding tubes, medicine administration, strategies

## Abstract

**What is known and objectives:**

The use of enteral tube feeding at home is becoming more widespread, with patients ranging in age and diseases. Dysphagia and swallowing difficulties can compromise nutritional intake and the administration of oral medications, affecting therapeutic outcomes negatively. Carers’ experiences of medicines administration and medicines optimization have not been explored fully. The objectives of this study were to identify issues carers experience in medicines administration; the strategies they have developed to cope; and suggestions to improve the medicines administration process.

**Methods:**

An online survey was promoted nationally; 42 carers completed it. Descriptive statistical analysis was applied, as well as thematic analysis of open‐ended responses. Results were compared against the 4 principles of medicines optimization.

**Results and discussion:**

93% of respondents administered medications with enteral feeding tubes, but only 62% had received advice from healthcare professionals and only 8% had received written information on how to do so. Responses identified 5 medicines administration issues experienced by carers; 4 strategies they developed to cope; and 3 main areas of suggestions to improve medicines administration via enteral feeding at home.

**What is new and conclusion:**

The 4 principles of medicines optimization have not previously been applied to enteral feeding. We present a novel account of carers’ experiences, for example coping with ill‐suited formulations and a lack of training and support, which should inform better practice (Principle 1). Carers sometimes experience suboptimal choice of medicines (Principle 2). Carers’ practices are not always well‐informed and may affect therapeutic outcomes and safety (Principle 3). There is scope for improvement in carer training, education and support to better support medicines optimization (Principle 4).

## Graphical abstract

Carers of patients using enteral tube feeding experience a variety of issues when administering medications, such as coping with ill‐suited formulations and a lack of training and support. Carers’ practices are not always well‐informed and may affect therapeutic outcomes and safety. There is scope for improvement in carer training, education and support to better support medicines optimization.

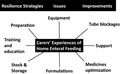

## 1 WHAT IS KNOWN AND OBJECTIVES

Nutrition and medicines support is critical for human well‐being. Swallowing difficulties can compromise nutritional intake and affect the administration of oral medicines. Dysphagia, or swallowing disorders, may develop as part of normal ageing or be caused by conditions such as Parkinson's disease, dementia and stroke.^1^ Feeding problems are also common in children.^2^ Dysphagia may require an enteral tube to receive nutrition and medicines. Enteral tube feeding at home has become more widespread.^3^, ^4^ It is indicated for a variety of reasons, ranging from dysphagia in older people to inadequate nutrition in children.^5^, ^6^ In 2010, 16 986 children and 31 776 adults were registered in the UK using home enteral tube feeding.^7^

Many practical issues may affect patient safety, including accidental or intentional tube dislodgement; pump inaccuracy; frequent tube blockages; inappropriate storage of feed, medicines and equipment, and night‐time carer sleep disturbance.^3^, ^8^, ^9^

Optimization of the use of medicines has been a focus in recent years.^10^ Four principles are proposed:

1. aim to understand the patient's experience;
2. provide an evidence based choice of medicines;
3. ensure medicines use is as safe as possible; and
4. make medicines optimization part of routine practice.

To our knowledge, these principles have not been applied to the administration of medication via enteral tubes in home care.

1. There is a need to understand carers’ experience to develop recommendations on how to make enteral medication administration easier, safer and more effective.
2. There is a need to understand the choice of medicines and the consequences of adapting medications for enteral tube delivery.
3. There is a need to explore the workarounds and informal practices carers develop to avoid error and cope with complex medication regimes in suboptimal circumstances^11^: adapting medications can cause concern to healthcare professionals (HCP) and carers.^12^, ^13^ Issues include the impact of modifying medicines on safety and efficacy profiles and legal implications of modifying medicines, due to unavailability of appropriate formulations.^13^, ^14^, ^15^, ^16^
4. There is a need to explore the support carers receive to ensure that medicines optimization is part of normal practice.

The few studies that have explored experiences of medicines administration and enteral feeding^17^, ^18^, ^19^ were not based in the home setting. Carers often have less knowledge and training than nurses, and the home has a different physical and social dynamic compared to care homes and hospitals. Furthermore, few previous studies have focused on carers’ perceptions on issues associated with enteral feeding in the home setting.^8^, ^20^

The purpose of this study was to explore carers’ experience of home enteral feeding for medicine administration, the strategies they develop, and their suggestions for improving medicine administration; we relate these to the principles of medicines optimization.

## 2 METHODS

### 2.1 Ethical approval

Ethical approval was obtained from University College London Research Ethics Committee (UCLIC/1213/015). Informed consent was obtained from all participants in the study.

### 2.2 Survey development

A review of the literature was conducted to gather initial data on carer strategies for home enteral feeding. A patient and public involvement advisory group was established to review and refine the survey, based on current best practice.^21^, ^22^ Three members were carers with experience of home enteral feeding, and the fourth was a parenteral nutrition patient who worked with the charity Patients on Intravenous and Nasogastric Nutrition Treatment (PINTT). The survey was implemented using Qualtrics and was divided into 9 sections: the respondent and who they care for; required training and support; day‐to‐day equipment use; pump and ancillaries; nutrition and hydration; administration of oral medicines via feeding tube; coping with the feeding regime while away from home; hints and tips about the use of feeding tubes; and respondents’ suggestions for making enteral feeding easier.

### 2.3 Data collection

The survey was promoted nationally through the PINTT website, PINTT quarterly magazine and social media outlets such as Twitter. The incentive of entering a prize draw was offered to participants who completed the questionnaire. Survey participants were required to be 18 years or over and to be carers, family or paid, of a person who needed assistance with enteral tube feeding and medicines administration. The survey was open for 2 months. The estimated time to complete the survey was an hour. Consent was obtained at the beginning of the survey. The survey was anonymous, with participants only required to provide personal details to be included in the prize draw.

### 2.4 Data analysis

Descriptive statistical analysis was conducted using SPSS version 21. Open‐ended responses were extracted from the survey and analysed thematically. Initial coding was conducted by DA, and a coding framework was developed to categorize themes and subthemes. Thematic analysis results were discussed within the research group. The 4 principles of medicines optimization were utilized in the final stage of analysis to relate results to the principles and inform the implications on practice.

## 3 RESULTS

Results are reported under 3 main themes: medicine administration issues, resilience strategies and suggestions for easier enteral tube use. Table 1 shows participant profiles.

**Table 1:** Respondent characteristics

Carer age range	Number of carers
18‐29	4
30‐39	12
40‐49	17
50‐59	6
60‐69	3
	Total: 42

Care recipient relationship	Number of care recipients
Parent	2
Grandparent	1
Son/daughter	31
Sibling	2
Partner/spouse	5
Non‐family member, for example service user	2
	Total: 43^a^

Care recipient age range	Number of care recipients
≤10	20
11‐18	11
19‐29	4
30‐39	2
40‐49	0
50‐59	2
60‐69	1
≥70	3
	Total: 43^a^

Reasons for enteral feeding tube placement	Number of care recipients (%)
Failure to thrive	7 (16)
Swallowing difficulties due to disability, stroke, brain injury	18 (42)
Issues to digestive system (eg Crohn's disease, gastroparesis)	18 (42)
	Total: 43^a^

a One respondent is taking care of 2 family members.

### 3.1 Medicines administration issues

A large proportion of respondents administer medication via feeding tubes without having received written information on how to do this. Most needed to administer solid oral dosage forms (SODF), and not all had received instructions on avoiding tube blockages (Table 2). The most common advice was to flush with water before and after the medicine, make sure the medicine is dissolved properly in warm water, or flush with water mixed with other ingredients, such as sodium bicarbonate.

**Table 2:** Medicine administration quantitative results

	Number of carers/total number of respondents (%)
Carers administering medicines in enteral feeding tube	
Yes	39/42 (93)
No	3/42 (7)
Received information on how to administer medicines	
Received written information‐haven't read it	1/39 (3)
Received written information and read it	2/39 (5)
Received advice from HCP	24/39 (62)
No	12/39 (31)
Received instructions on tube blockages and how to prevent them	
Yes	25/39 (64)
No	14/39 (36)
Administer medicines that are available as solid oral dosage form	
Yes	26/39 (67)
No	13/39 (33)
Dilute liquid medicines or add them to enteral feed	
Yes	8/39 (21)
No	31/39 (79)
Have concerns or difficulties about administering medicines	
Yes	5/39 (13)
No	34/39 (87)

Five issues relating to medicines administration were identified (Table 3). A wide variety of medicines and dosage forms were administered. Medicines were not always available in the form required for easy and safe administration. This caused carers to *modify medicines*, such as crushing tablets. Liquid medicines were advocated to overcome problems associated with SODF; however, some carers reported having to dilute liquids that were too viscous. Modifying SODF may lead to the medicine blocking the tube, and carers had to find ways to prevent this. Proton pump inhibitors (PPI) were associated with the most tube blockage issues.

**Table 3:** Medicines administration issues (qualitative results)

Medicines administration issue	Description of issue	Quotes to illustrate issue
1. Number and variety of medicines	Wide variety and large number of medicines can complicate administration, be a length process, and be uncomfortable for the care recipient.	“Over the years a range. All in a liquid or dissolvable form Ventolin (salbutamol), Epilim (sodium valproate), paracetamol, antibiotics, ibuprofen, gabapentin, codeine, Imodium (loperamide) and probably others!” (Carer 39)
		“She takes a lot of medications that are syringes in one after another and can be a lot in one go and make her feel sick”. (Carer 5)
2. Inappropriate formulations	Medicines were not always in a suitable form for safe and effective administration.	“Many meds were administered. Liquid was given which was fine. Pills were given, which we would mix with lukewarm water so it diluted the pill. Tablets were crushed and put into lukewarm water. Some tablets with a plastic coating we tipped out the contents and throw away the outside. We were given permission to do this by doctors”. (Carer 40)
3. Liquid formulations were not always ideal	Liquid formulations may be viscous and thus cause tube blockages	“Just dilute Peptac (calcium carbonate, sodium bicarbonate, sodium alginate) because it's so viscous and thick and hard to push down the tube. We draw up the 10 mL then draw a further 5 mL into syringe and agitate it to mix.” (Carer 3)
4. Modifying formulations	Inappropriate formulations led carers to modify formulations, sometimes without advice. Modifications may lead to tube blockages	“Crush tablets in tablet crushers, add water to tab crusher bowl wait until dissolved, draw up into syringe and administer through tube. Have had massive problems with Zoton (lansoprazole) blocking tubes in the past. Must do largish flush before and after administering, and dissolve it in full 10 mL water and move syringe around whilst administering to stop it lumping together.” (Carer 3)
5. Difficulty obtaining suitable formulation	Obtaining appropriate formulations was not always easy for carers	“I have had to fight for liquid forms of melatonin + omeprazole to stop blocking her mickey.” (Carer 4)

### 3.2 Resilience strategies

Four main strategies to make medicines administration easier and reduce the likelihood of problems occurring were identified (Table 4). Trying to prevent medicines from causing tube blockages required carers to develop and try different strategies.

**Table 4:** Resilience strategies developed by carers

Description of strategy	Quotes to illustrate strategy
1. General strategies were developed when administering medicines and feeds to make the process easier, such as the practicalities with storage and medicine preparation	“Put the tablets in the syringe and draw water in and leave it to dissolve for half an hour‐much easier than crushing and mixing with water and trying to draw it all into a syringe. Use a 60 mL syringe of water and using part of it 3 times instead of lots of 10 mL syringes of water. Sitting the plungers of the 60 mL syringes in water so they don't stick. Always removing air before meds.” (Carer 2)
	“We store the connector and giving set in a tub in the fridge. Everything is always stored in the same place. We have done a photo timetable of our own to show step by step instructions of feeding instructions to give to relatives/school.” (Carer 10)
2. Preparing medicines in syringes in advance	“I prepare a day's feed and medicines the night before and store in the fridge I make sure everything I need for a full day is stored together, so nothing is forgotten and I have everything I need at hand” (Carer 30)
	“Prepare giving sets and any medicines in advance especially when going out. Ready filled medicine syringes are easier to transport for day trips rather than huge medicine bottles.” (Carer 35)
3. Carers developed strategies to dissolve feeds and medications, such as mixing with boiled water	“I use hot boiled water to make the feeds as I find it dissolves the formula/powder better than cooled water does I use boiling water to dissolve his Nalcrom (sodium cromoglycate) tablet to ensure it dissolves fully and mix with his other meds to administer them all together” (Carer 30)
4.Carers developed ways to prevent and/or deal with tube blockages such as using fizzy drinks or juice and shaking syringes to aid dissolving process	“Cola works wonders for a blocked tube.” (Carer 19)
	“Shake dissolved tablets, so shake the syringe as you push to stop blockages.” (Carer 12)
	“We were advised to give with extra water, this didn't work so we were told to mixed it with sodium bicarbonate which didn't work, we used a liquid version but this was too expensive and Drs refused to prescribe it any more so we learnt just to follow with a big flush immediately.” (Carer 41)

### 3.3 Suggestions for easier enteral tube use

Carers provided suggestions for easier medicines administration and enteral tube use that related to the wider context of equipment, training, support and medicine formulations (Table 5).

**Table 5:** Suggestions to make enteral tube use easier for carers

Suggestions made by carers	Quotes to illustrate suggestion
Improving equipment such as making the tubes more aesthetic, designing kid‐friendly equipment, and providing better quality of tubes	“The quality and endurance of the tubes themselves, the smell of the feeds and the useless rucksacks that are utterly unsuitable and ugly for kids and quieter pumps that disturb all the children in the classroom.” (Carer 6)
	“The only thing I can think of is to be able to carry the pump and feed in a handbag instead of a backpack. It's quite awkward when attending special occasions eg weddings, christenings, funerals and such like” (Carer 26)
The availability of appropriate formulations, such as liquids	“Have all meds available in liquid form ‐ would save me a lot of time, counting tablets, crushing them ensuring everything goes into syringe and then washing 5 tablet crushers and lids per day.” (Carer 3)
Improving training and support Written instructions, which include a trouble shooting page Basic training as well as training on dealing with problems Being able to practice under supervision rather than just observing the nurses More person‐centred training rather than equipment centred training Ensuring there is an appointment with nurses at home before discharge 24‐h support in early days after discharge Having a single point of contact rather than being passed from person to person Out of hours service should be empathetic, helpful and welcoming	“Let users tinker with prototype of the feeding pump at home to find out stupid design flaws.” (Carer 13)
	“Good knowledge and expertise. Having face to face contact with health professionals when unforeseen problems occur, especially during the night, bank holidays, weekends” (Carer 20)
	“Have a good proactive and friendly enteral team ‐ approachable and able to offer holistic practical advice to parents ‐ without prejudice! We haven't got that in our area anymore and it makes me more aware how much better it was years ago! I have 10 y of tube feeding experience ‐ and have no one to call on for help! Terrifying and you just have to hope everything goes OK” (Carer 27)

## 4 DISCUSSION

Home enteral tube use is challenging; this study identified difficulties faced by carers in daily life when administering medicines through enteral tubes and how this process can be optimized to meet patient needs. The results also shed light on carer practices that may negatively affect medicines use.

An important issue was the modification of medicines for easier use, with carers diluting liquids and crushing and dissolving SODF. This highlights various concerns: the unavailability of appropriate dosage forms; legal implications of modifying medicines; consequences of altering medicines and mixing them together or with feeds; and the lack of information aimed at carers regarding effective medicines use. In addition, the co‐administration of medicines with feeds may affect the bioavailability of the medicines, raise compatibility issues and cause interactions.^23^, ^24^, ^25^

Some respondents specified which medicines they had difficulties with and how they overcame these difficulties. Carers who administered PPIs, such as omeprazole or lansoprazole, all faced tube blockage complications; tube blockages are one of the most common complications encountered with enteral tubes, and lansoprazole is known to cause blockages.^6^ This class of medicines is acid labile, so their effectiveness is compromised when they come into contact with gastric acid.^24^ Some PPI formulations, such as lansoprazole gastro‐resistant capsules, contain enteric coated granules, which can be mixed with water or apple juice before administration through a nasogastric tube,^26^ allowing the medicine to pass through the stomach and be released in the duodenum. However, this formulation still does not guarantee that blockages will not occur in the feeding tube. Some studies have shown that mixing PPIs with an alkaline bicarbonate solution is safe, effective and less likely to cause blockages than mixing with juice.^27^, ^28^, ^29^, ^30^

Liquid formulations are often preferred over SODF for administration through enteral tubes. Unfortunately, the particle size of drugs in suspension, even when diluted, may still cause occlusion of tubes, as exemplified by the use of ciprofloxacin suspension by 1 respondent. Other disadvantages include the potential for drug instability due to hydrolysis and oxidation and the viscosity of suspensions causing difficulties with administration.^23^ Liquid medicines are also more expensive than SODF, and some HCPs may be reluctant to prescribe them. Also, 8 respondents reported diluting liquid medicines to decrease their viscosity. For example, 1 carer mixed calcium carbonate with feeds for administration; the calcium might bind with the phosphate in the feed.^15^ This highlights the need for more appropriate dosage forms and formulations for use with enteral feeding tubes.

Polypharmacy also creates a challenge for carers and poses a risk with enteral tubes. Some carers administered medicines together which might increase the risk of drug‐drug interactions and compromise the effectiveness of the medicines.^23^ The process of administering medicines was also seen as being lengthy by some carers. To optimize medicines use and reduce carer burden, medicines should be reviewed and rationalized to remove any unneeded medicines and where possible to reduce the frequency of medicines administration.

Proper tube care and correct flushing methods should be included in training for carers, as tube blockages may occur due to inappropriate medication preparation, interactions between medicines and feeds, and incorrect flushing procedures.^15^ These practices can cause complications; the use of carbonated or acidic drinks might denature the contents of the feed and/or the medicine.^31^ The adsorption of fluids used to unblock tubes can pose a risk of interacting with medicine administered later. Various methods of clearing enteral feeding tube occlusions have been proposed; however, there is no consensus on the best approach.^32^

Medicine dosage and efficacy may be negatively affected by practical methods developed by carers, such as preparing medicine syringes in advance. This is a greater issue for medicines with a narrow therapeutic index, such as antipsychotic drugs. Respondents also reported some risky ways of modifying medicines, including dissolving tablets in boiling water. Such practices can cause significant changes to the physicochemical properties of the drug and hence the therapeutic outcome for the patient. The majority of respondents (87%) did not have concerns about the way medicines are given through the tube (eg avoiding mixing certain fluids or changing the form of the medicine), suggesting a lack of awareness of risks associated with the administration of modified medicines.

Carers reported a wealth of resilience strategies; although not all would be considered appropriate or safe, they illustrate ways carers overcome challenges and highlight problems that HCPs may not be aware of. This should be further explored, to develop patient/carer information to share tips and tricks, and also highlight risks, for example using soda to unblock tubes.

This highlights the need for formulations that can be administered without the need for modification, as well as better education on safe and effective methods to administer medicines. It has also been reported that some pharmacists possess limited knowledge regarding safe administration of medicines via enteral feeding.^33^ Training and support should be consistent, meet carers’ priorities and take into account their experience with using enteral feeding pumps.

There is also an opportunity to develop products that are compatible, easy and safe to use. The development of patient‐centric medicines should involve a team of formulation scientists and medical device experts to ensure compatibility between the drug formulation and the administration device.^15^, ^16^, ^24^ Pharmaceutical companies are encouraged to provide information on safe administration and/or modification of medications where administration of a preparation through a feeding tube is considered to be very likely.^34^

### 4.1 Limitations

Recruitment was greatly assisted by PINNT, which may affect the generalizability of the findings. The majority of the participants were middle‐aged carers providing care for their children. Bias may have been introduced because people who have had medication administration difficulties might have been more motivated to respond. This may not be a representative sample as most people with enteral tube feeding are older people,^7^, ^35^ but it still highlights issues from a key patient population.

## 5 WHAT IS NEW AND CONCLUSION

Applying the 4 principles of medicines optimization to carers’ experiences of administering medicines via enteral tubes, we have identified challenges faced by carers, the potential compromise in clinical care in people using home enteral feeding, and the strategies they use to cope in suboptimal circumstances (Principle 1). The data show that the selection of medicines can cause problems for carers who need to administer these formulations (Principle 2). Little was previously known about potential errors and solutions adopted to overcome practical difficulties that occur in the home; this study has highlighted inappropriate practices adopted by carers and their potential impact on therapeutic outcomes (Principle 3). Furthermore, respondents reported a lack of written instruction for administering medications via this route or how to avoid tube blockages, and identified areas of improvement for training and support (Principle 4).

There is a need for further research in this area, to deliver safe practice recommendations, improved training of HCPs and carers and standardized information for carers and patients.

## CONFLICTS OF INTEREST

The authors declare that they have no conflict of interest.
